# Supplementary material for: DMRforPairs: identifying Differentially Methylated Regions between unique samples using array based methylation profiles
Source: BMC Bioinformatics. 2014 May 15;15:141. doi: 10.1186/1471-2105-15-141 (PMC4046028; doi:10.1186/1471-2105-15-141)
Supplement: Additional file 2 — DMRforPairs output for the comparison of A431-MCF7 and NA17018-NA17105. Please start from the HTML files in each folder. Available via the BMC Bioinformatics website. [file 1471-2105-15-141-S2.zip › 1394847754114233_MOESM2_ESM/A431_MCF7/figures/18765.pdf]

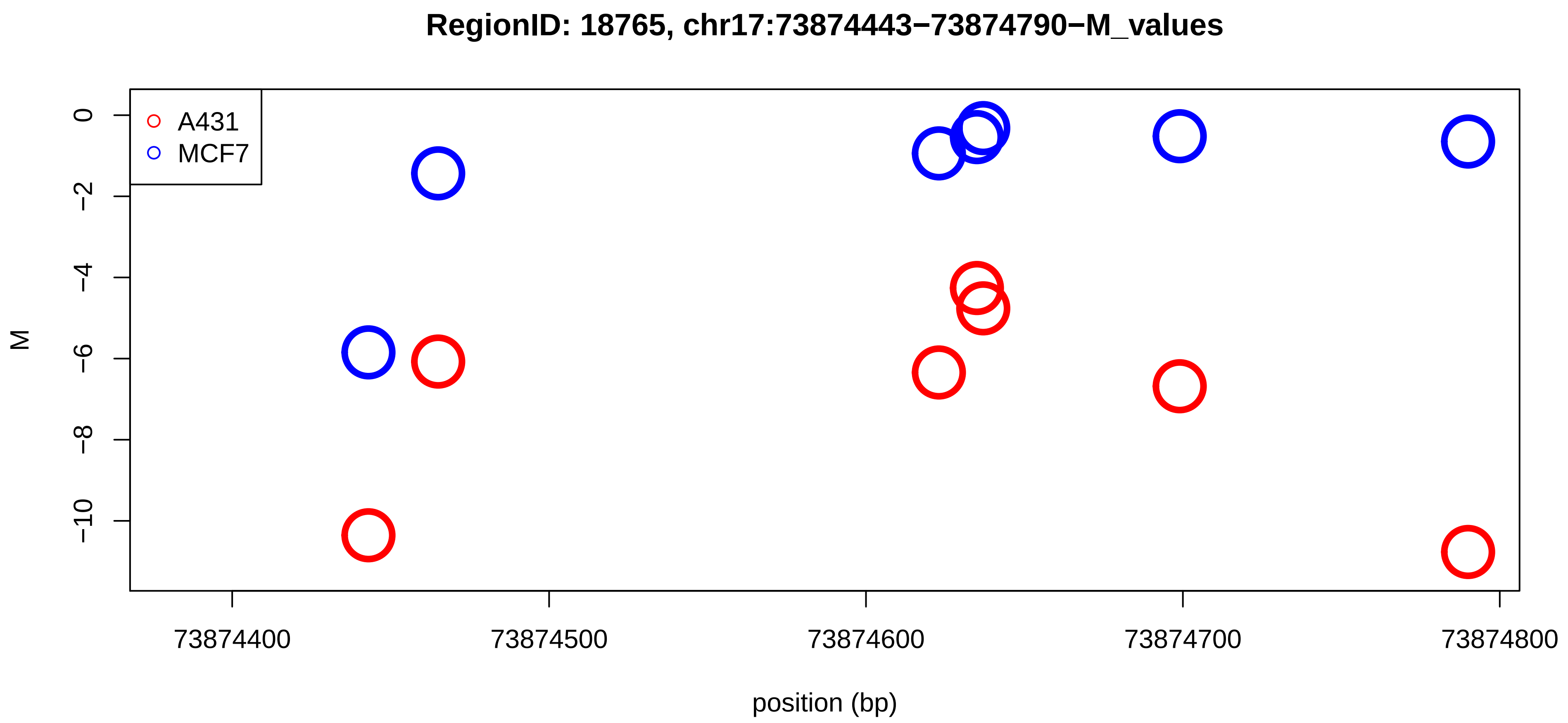
RegionID: 18765, chr17:73874443−73874790−M_values
A431
MCF7
M
0
−2
−4
−6
−8
−10
73874400
73874500
73874600
73874700
73874800
position (bp)

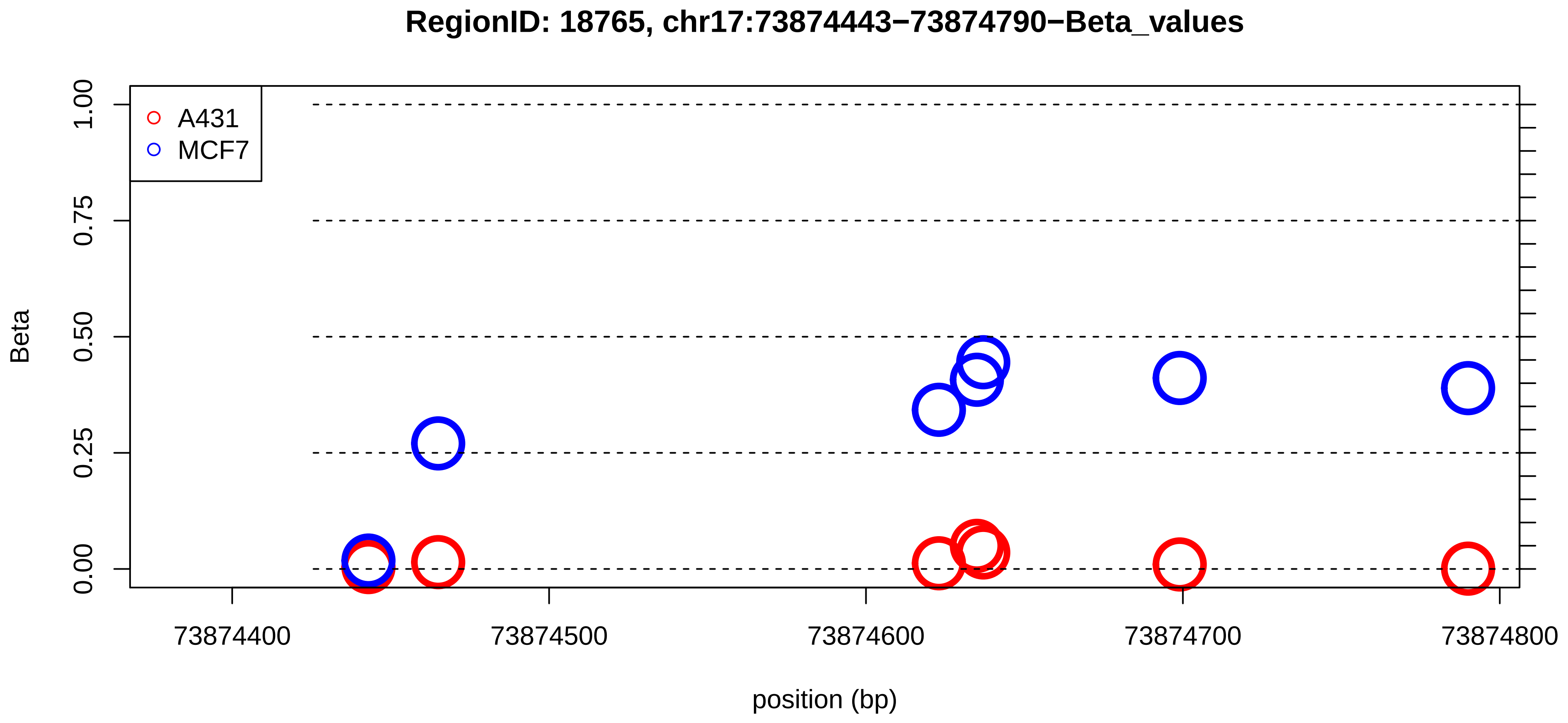
RegionID: 18765, chr17:73874443−73874790−Beta_values
A431
MCF7
Beta
1.00
0.75
0.50
0.25
0.00
73874400
73874500
73874600
73874700
73874800
position (bp)

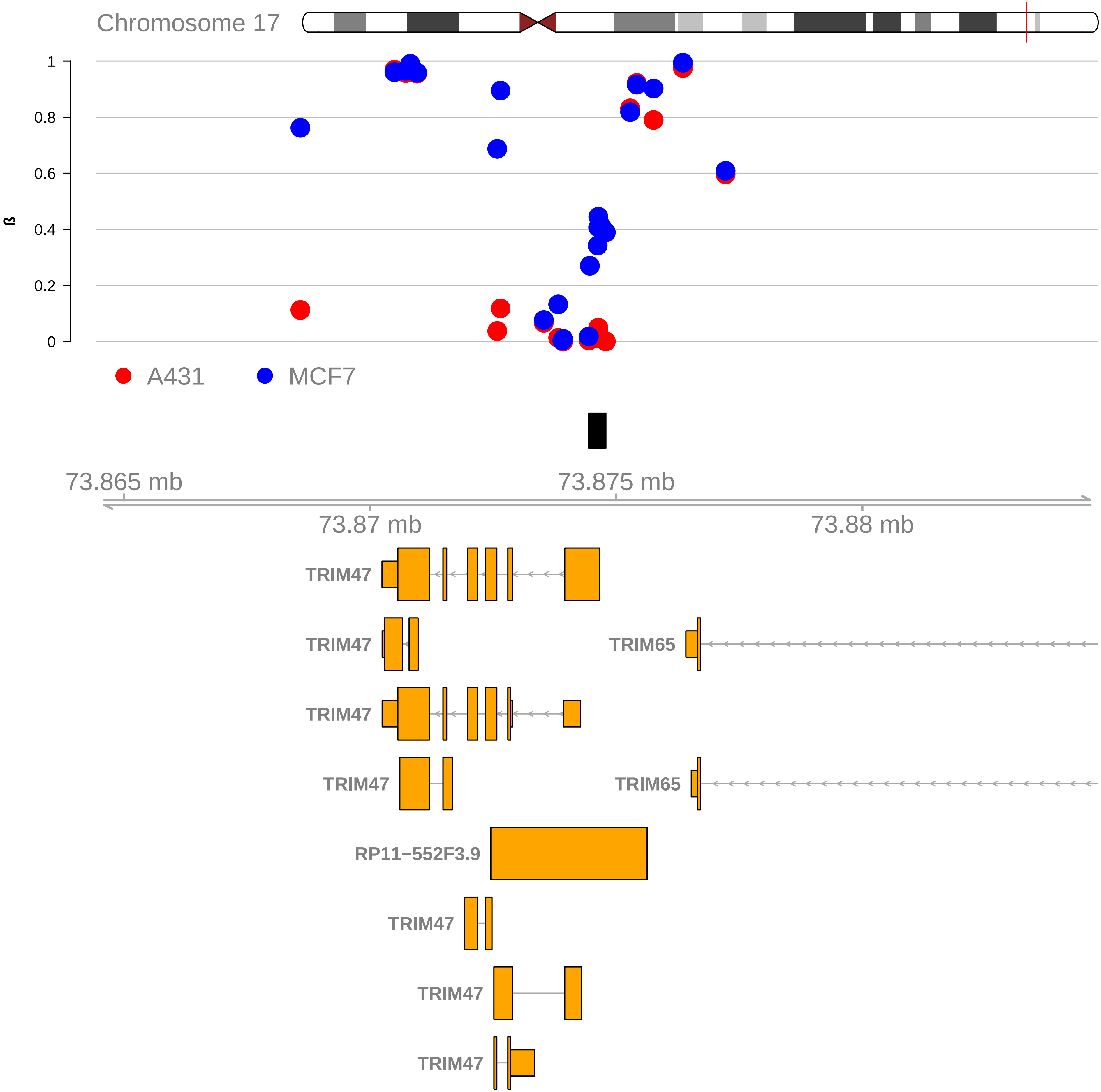
Chromosome 17
ß
1
0.8
0.6
0.4
0.2
0
A431
MCF7
73.865 mb
73.875 mb
73.87 mb
73.88 mb
TRIM47
TRIM47
TRIM65
TRIM47
TRIM47
TRIM65
RP11−552F3.9
TRIM47
TRIM47
TRIM47
